# Atomically-precise colloidal nanoparticles of cerium dioxide

**DOI:** 10.1038/s41467-017-01672-4

**Published:** 2017-11-13

**Authors:** Kylie J. Mitchell, Khalil A. Abboud, George Christou

**Affiliations:** Department of Chemistry, University of Florida, Gainesville, FL 32611-7200 USA

## Abstract

Synthesis of truly monodisperse nanoparticles and their structural characterization to atomic precision are important challenges in nanoscience. Success has recently been achieved for metal nanoparticles, particularly Au, with diameters up to 3 nm, the size regime referred to as nanoclusters. In contrast, families of atomically precise metal oxide nanoparticles are currently lacking, but would have a major impact since metal oxides are of widespread importance for their magnetic, catalytic and other properties. One such material is colloidal CeO_2_ (ceria), whose applications include catalysis, new energy technologies, photochemistry, and medicine, among others. Here we report a family of atomically precise ceria nanoclusters with ultra-small dimensions up to ~1.6 nm (~100 core atoms). X-ray crystallography confirms they have the fluorite structure of bulk CeO_2_, and identifies surface features, H^+^ binding sites, Ce^3+^ locations, and O vacancies on (100) facets. Monodisperse ceria nanoclusters now permit investigation of their properties as a function of exact size, surface morphology, and Ce^3+^:Ce^4+^ composition.

## Abstract (web summary)

Control over nanoparticle atomicity has not yet been extended to metal oxides. Here, the authors present the synthesis and X-ray crystal structures of a family of atomically precise ceria nanoclusters, a pivotal step for understanding the relationship between their atomic structure and reactivity.

## Introduction

Since its introduction in 1976 as an oxygen-storage component to ensure the efficient activity of the noble metals used in three-way catalysis in automobile exhausts^1–3^, cerium(IV) dioxide (CeO_2_, ceria) has become of considerable utility as a catalyst or co-catalyst in industrial, petrochemical and environmental processes^2–7^. In addition, CeO_2_-containing materials are often used in oxide fuel cells^8^, precision polishing materials^9,10^, UV filters^10^, corrosion prevention^11^, and other applications^1,12,13^.This widespread use of Ce is partially due to its significant abundance (0.0046% by weight of the Earth’s crust) and its Ce^3+^/Ce^4+^ redox couple, which is crucial to many applications by facilitating the formation of CeO_2−*x*_, containing highly reactive defect sites comprising O vacancies and attendant Ce^3+^ ions^1,12,14^. Ceria can thus act as an efficient oxygen buffer, assisted by oxygen mobility within its layered fluorite structure. In fact, bulk ceria naturally contains relatively few Ce^3+^/O-vacancy defect sites at ambient temperatures, but their number increases at higher temperatures where Ce^4+^ reduction and oxygen release are favoured. Catalysis by bulk ceria is therefore normally carried out at temperatures > 450 °C.

In the last decade, interest in ceria nanoparticles (CNPs) has seen explosive growth due to their much greater reactivity and increased catalytic efficiencies at lower temperatures^9,12^. Significant CNP activity at or near room temperature has also been established^15^, as has facet-dependent reactivity^12,16^. For example, appreciable oxygen storage capacity is observed at 150 °C on the cubic (100) face of nanoceria crystals, which is ~250 °C lower than for irregularly shaped nanoceria or the bulk material^17^. CNPs are also under investigation as photovoltaic materials in solar cells whereas bulk CeO_2_ has no photovoltaic response^18^. Using CNPs instead of a cerium oxide support increases by two orders of magnitude the activity of a Au catalyst for the selective oxidation of CO^19^. In addition, the higher reactivity of CNPs at ambient temperatures is permitting many important biomedical applications to be developed, such as scavenging of reactive oxygen species (ROS)^12,20–22^. The CNP activity and toxicity to living tissue clearly depend on particle size and surface composition (e.g., Ce^3+^/Ce^4+^ ratios), but as is normally the case in all areas of nanoparticle science, the problems of polydispersity, agglomeration, and surface variations have plagued detailed study of these parameters^10,23,24^. For CNPs, it is particularly challenging to determine the concentration and locations of Ce^3+^, attendant O vacancies, and protonated O (i.e., OH^−^, H_2_O) species on the surface, and the relationship between them^10^. A more controlled approach to monodisperse CeO_2_ nanoclusters and nanoparticles is clearly needed, especially at the ultra-small, sub-20 nm sizes that are of growing importance, particularly for biomedical applications.

We now describe development of procedures using simple Ce^4+^ salts and organic reagents that yield a family of monodisperse ceria nanoclusters of different sizes depending on the carboxylic acid employed. Such an approach was recently accomplished for monodisperse metal nanoclusters, primarily of Au, stabilized by thiolate ligands^25,26^. In our work, the ligands of choice for metal oxide nanoclusters are carboxylates, especially since oleic and similar acids are common surfactants in metal oxide nanoparticle syntheses^20,27^. The solubility and monodisperse nature of the products we obtain allows molecular crystals to be grown, leading to structural characterization of the nanoclusters and their surface features to atomic precision by single-crystal X-ray diffractometry. The nanoclusters are [Ce_24_O_28_(OH)_8_(PhCO_2_)_30_(py)_4_] (**1**; Ce_24_), [Ce_38_O_54_(OH)_8_(EtCO_2_)_36_(py)_8_] (**2**; Ce_38_) and [Ce_40_O_56_(OH)_2_(MeCO_2_)_44_(MeCO_2_H)_2_(py)_4_]/[Ce_40_O_56_(OH)_2_(MeCO_2_)_44_(MeCN)_2_(py)_4_] (**3a/b**; Ce_40_), where py is pyridine. **3a**/**3b** denote the two independent nanoclusters in the asymmetric unit of **3**, which differ slightly in the organic ligation. **2** also contains two independent nanoclusters (**2a**/**2b**), but they have the same formulation.

## Results

### Nanocluster structures

**Fig. 1:**
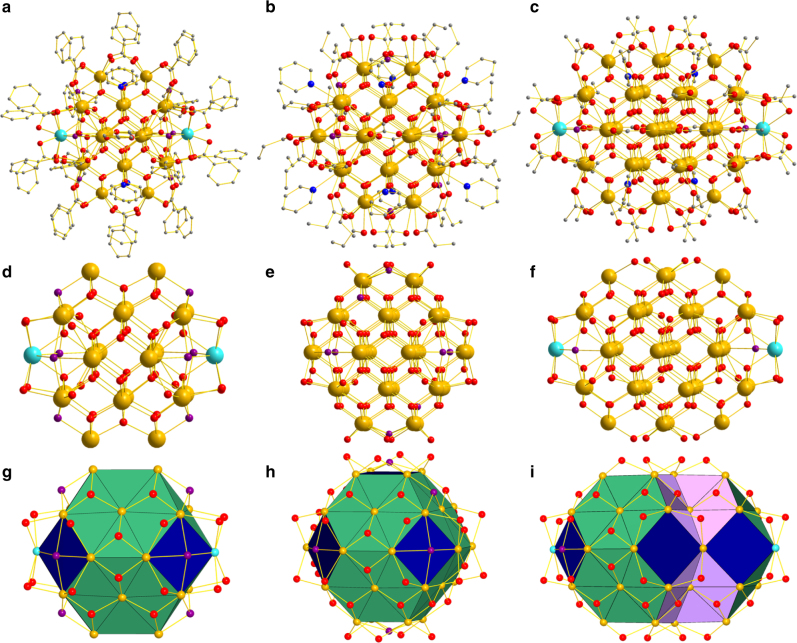
Structures of ceria nanoclusters. **a**–**c** show the complete structures of **1** (Ce_24_), **2**(Ce_38_), and **3a** (Ce_40_), respectively. H atoms have been omitted for clarity. Atom sizes of C, N, and O are made small to emphasize Ce locations. Colour code: Ce^IV^ gold, Ce^III^ sky blue, O red, N blue, C grey. **d**–**f** show their Ce/O cores from the same viewpoint (including carboxylate O atoms that are bridging) using the same colour code except that protonated O atoms (i.e., OH^−^ ions) are indicated in purple. **g**–**i** show the cores again, from approximately the same viewpoint but with surface facets colour-coded: (100) facets are blue; (110) facets are violet; (111) facets are green. Only carboxylate O atoms that are bridging are included

Several pertinent points about the complete structures of **1**–**3** (Fig. 1a–c) will be summarized to allow for convenient comparisons. They all comprise Ce_*x*_O_*y*_ cores (excluding carboxylate O atoms) with metal (*x*) and total (*x* + *y*) atom counts of 24/60, 38/100 and 40/98 for **1**–**3**, respectively, and they exhibit the same fluorite structure as bulk CeO_2_, i.e., alternating layers of 8-coordinate cubic Ce^4+^ and 4-coordinate tetrahedral O^2−^ions. Some surface Ce^4+^ ions are 7- or 9-coordinate (vide infra). From the viewpoint of Fig. 1d–f, the cores consist of five Ce layers in an A:B:C:B:A pattern (**1**, *A* = 2, *B* = 6, *C* = 8; **2**, *A* = 4, *B* = 9, *C* = 12; **3**, *A* = 4, *B* = 10, *C* = 12), leading to the Ce_38_ core of **2** being essentially spherical (1.12 × 1.12 × 1.12 nm) whereas those of Ce_24_ (**1**, 0.75 × 1.10 × 1.10 nm) and Ce_40_ (**3**, 1.13 × 1.13 × 1.61 nm) are ellipsoidal. The Ce_38_ can also be described as a ‘truncated octahedron’, a structure that is one of those recently predicted by DFT studies to be favoured for Ce_*x*_O_*y*_ fragments of CeO_2_ in this size range^28^. **2** contains only Ce^4+^, but **1** and **3** each also contain two 10-coordinate Ce^3+^ ions at opposite ends of the cores, as suggested by DFT calculations on Ce^3+^ in Ce_*x*_O_*y*_ fragments of CeO_2_^28,29^. The Ce oxidation states were confirmed by bond valence sum (BVS) calculations (Supplementary Table 2) and the detection of Ce^3+^ (*S* = ½) EPR spectra for **1** and **3**. The latter were measured on microcrystalline powders at 5 K (Supplementary Figs. 1 and 2) and are comparable with the few Ce^3+^ EPR spectra reported for CeO_2_ nanoparticles or Ce^3+^ doped into polymeric species^30–32^. Nanoclusters **1**–**3** are large enough to display multiple facets (Fig. 1g–i), as do CNPs; the different faces for CeO_2_ and Ce_40_ are defined in Supplementary Fig. 3. **1** and **2** display only (100) and (111) facets, whereas **3** exhibits these and also (110) facets. Finally, the cores are enveloped within monolayer organic shells of carboxylate and py groups, which exhibit only minor positional disorder in some C and O atoms (Fig. 1a–c).

The structural results thus strongly support the description of **1**–**3** as atomically precise ceria nanoclusters in the ultra-small size range corresponding to the smallest CNPs synthesized to date, and stabilized to agglomeration by the organic monolayers. CNPs at this sub-20 nm size are being heavily targeted for use in various applications, especially in the biomedical field because they show enhanced catalytic activity and regenerative properties^21,33,34^. **1**–**3** are larger than the few previously known Ce/O molecular species, most of which are Ce_6_ species^35,36^ and some with tridentate amino-alcohol N,O,O-chelates^37^. It should be noted that the large family of monodisperse, crystalline polyoxometalates, some with very high metal nuclearities and sizes approaching 4 nm, have been known for many decades, but they do not possess the structure of bulk metal oxides and therefore cannot be described as their nanoparticles.

### Surface features

X-ray crystallography has allowed definition to atomic resolution of the surfaces, which are crucial to CNP reactivity. The overall question is how the geometry and environments of surface Ce and O atoms differ from those of body atoms. Indeed, surface Ce^4+^ geometries in **1**–**3** differ markedly from the 8-coordinate cubic of body Ce^4+^ ions. Even those still 8-coordinate are significantly distorted, while many are 9-coordinate and there is even rare 7-coordination for Ce12/Ce22 in **3a/3b**, respectively; coordination numbers are listed for all Ce atoms in Supplementary Table 2. This variety reflects both the greater degrees of freedom at the surface and at the carboxylate ligation. Nevertheless, all body and surface Ce atoms are essentially at the positions they would occupy in bulk CeO_2_, as shown by the overlays in Supplementary Fig. 4. The larger nanoclusters Ce_38_ (**2**) and Ce_40_ (**3**) show very little deviation of Ce and O atoms from their positions in bulk ceria; the smallest, Ce_24_ (**1**), appears more pliable by showing greater deviation, but it is still small. Thus, the Ce_*x*_O_*y*_ cores of **1**–**3** really can be described as fragments of bulk ceria, stabilized/passivated by the monolayer of carboxylate and pyridine ligands.

**Fig. 2:**
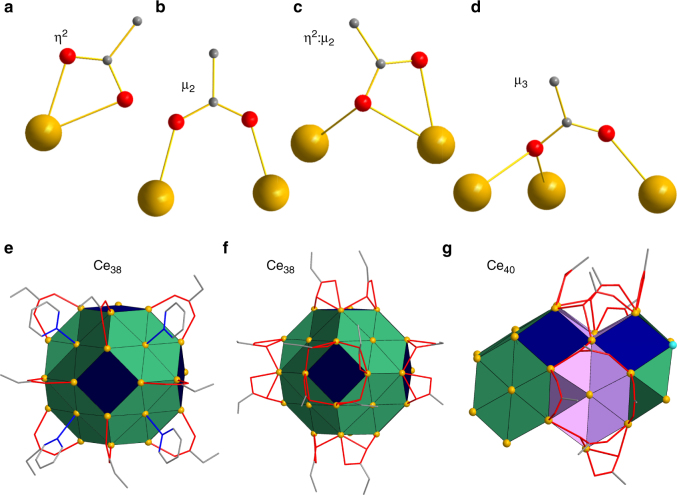
Ligand binding modes on the surface of ceria nanoclusters. The different binding modes of surface carboxylate and pyridine groups in **1**–**3**: **a** chelating (*η* ^2^); **b** µ_2_-bridging; **c** *η* ^2^-chelating and µ_2_-bridging; **d** µ_3_-bridging; **e** Ce_38_ (**2**) showing terminal pyridines ‘capping’ (binding to the center of) the (111) hexagons, and µ_2_-carboxylates bridging edges joining two (111) facets; **f** Ce_38_ (**2**) showing *η* ^2^:μ_2_-carboxylates at edges joining (100) and (111) facets; and **g** Ce_40_ (**3**) showing *η* ^2^:μ_2_- and μ_3_-carboxylates on edges of (110) facets, and µ_2_-carboxylates bridging edges joining (110) and (111) facets. Colour code: Ce^IV^ gold, Ce^III^ sky-blue, O red, N blue, C grey, (100) facets dark blue; (110) facets violet; (111) facets green

There are four types of carboxylate binding in **1**–**3** (Fig. 2a–d): chelating (*η* ^2^) and three doubly or triply bridging modes, allowing for flexibility and versatility in binding to one, two, or V-shaped sets of three surface Ce ions. The carboxylates can thus accommodate the multi-faceted surface structure, including points of high curvature (Supplementary Fig. 5), with terminal py groups completing ligation where necessary. Both types of μ_2_-carboxylates occur in all three nanoclusters and bridge Ce_2_ edges joining two facets, one of which is always a (111) facet (Table 1 and Fig. 2e–g). Interestingly, the *η* ^2^:μ_2_ mode is found only at (100)(111) and (110)(111) edges, whereas the μ_2_ mode is found only at (111)(111) and (110)(111) edges. In contrast, μ_3_-carboxylates occur only in **3**, bridging a V-shaped edge of the (110) facets. The *η* ^2^-chelating mode is also only found in **3**, always bound to one Ce of a (100) Ce_4_ square (vide infra). Terminal py ligands occur in all three nanoclusters, always ‘capping’ a (111) hexagon, i.e., attached to its central Ce (Fig. 2e). The two independent Ce_38_ nanoclusters in **2** are identical in formula and structure, but the two Ce_40_ units in **3** provide the benefit of slightly differing organic monolayer shells, revealing one way the latter can vary for a given nanocluster core. Thus, the chelating carboxylates (Fig. 2a) on two Ce^4+^ ions (Ce9) in **3a** are each replaced by a terminal MeCN (on Ce32) in **3b**, converting 9-coordinate Ce9 into 8-coordinate Ce32.

**Table 1:** Type of surface ligands in nanoclusters 1–3

Type	Binding mode	Found	Surface location
O^2−^	μ_3_-bridging	**1**–**3**	(111) or (110) Ce_3_ triangle
OH^−^	μ_3_-bridging	**1**, **2**	(111) Ce_3_ triangle
OH^−^	μ_4_-bridging	**1**–**3**	Lid on (100) Ce_4_ square
py	terminal	**1**–**3**	Capping of (111) hexagon
MeCN	terminal	**3b**	Lid on (100) Ce_4_ square
RCO_2_^−^	*η*_2_-chelating	**3**	Lid on (100) Ce_4_ square
RCO_2_^−^	*η* ^2^:μ_2_-chel/brid	**1**–**3**	Ce_2_ edge joining (100) (111)
		**3**	Ce_2_ edge joining (110) (111)
RCO_2_^−^	μ_2_-bridging	**1**–**3**	Ce_2_ edge joining (111) (111)
		**3**	Ce_2_ edge joining (110) (111)
RCO_2_^−^	μ_3_-bridging	**3**	V-shaped Ce_3_ edge of (110)

**Fig. 3:**
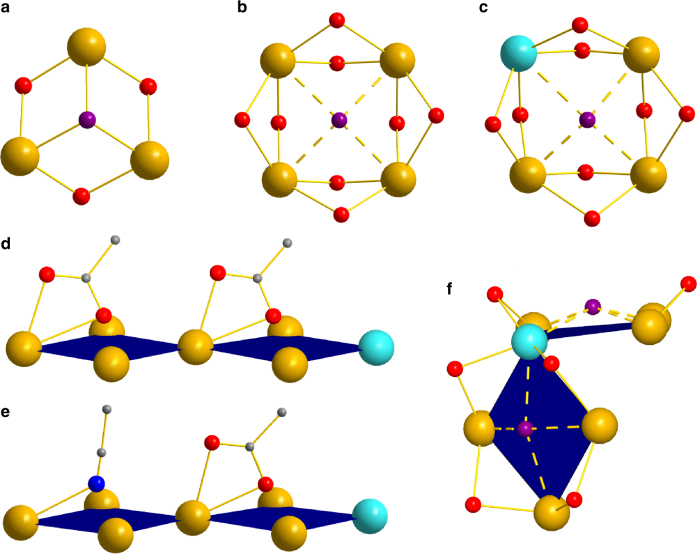
Structural features on the surface of ceria nanoclusters. **a** μ_3_-OH^−^ on (111) Ce^IV^_3_ triangle; **b** μ_4_-OH^−^ on a (100) Ce^IV^_4_ square; **c** μ_4_-OH^−^ on a (100) Ce^III^Ce^IV^_3_ square; **d** *η* ^2^-carboxylates in **3a** acting as lids on adjacent (100) Ce^IV^_4_ and Ce^III^Ce^IV^_3_ squares; **e** the analogous situation in **3b** to that in **d**, with an MeCN ligand replacing one *η* ^2^-carboxylate as lid; and **f** μ_4_-OH^−^ lids on a V-shaped (100) Ce^III^Ce^IV^_3_double-square in **1** linked at the Ce^III^corner. Color code: Ce^IV^ gold, Ce^III^ sky-blue, O red, OH^−^ purple, N blue, C grey. H atoms have been omitted for clarity

There are two distinct Ce surface subunits in **1**–**3** resulting from the fluorite structure, Ce_3_ triangles and Ce_4_ squares, and these will be described in turn. Ce_3_ triangles are very common surface units in (111) and (110) facets and are bridged primarily by pyramidal μ_3_-O^2-^ ions (Table 1), from tetrahedral body O^2−^ ions now binding one less Ce. Some in **1** and **2** are instead bridged by μ_3_-OH^−^ ions (Fig. 3a): The four in **1** are obvious from their O-H···N hydrogen bonding to lattice py molecules (O···N = 2.7–2.9 Å), which thus anchors the H^+^ on O15 and O16 and gives the expected O BVS of 1.21 (Supplementary Table 3). In contrast, the two μ_3_-OH^−^ in **2** are disordered since there is no reason for H^+^ to favour particular μ_3_-O^2−^ ions when so many are essentially equivalent. Slightly lowered BVS values (1.52–1.72) for the four μ_3_-O^2−^ ions at O18/O39 and the four at O49/O60 in **2a** and **2b**, respectively (Supplementary Table 4), suggest that the 2H^+^ are randomly distributed primarily among these positions to give partial μ_3_-OH^−^ occupancies.

In body Ce_4_ squares, each edge is oxide-bridged, but at the surface the edges are carboxylate-bridged. These are the (100) facets (Fig. 1g–i) and occur in three slightly different forms. The six separated Ce^4+^_4_ squares in **2** (Fig. 3b), the two Ce^4+^_3_Ce^3+^Ce^4+^_3_ V-shaped double-squares fused at a Ce^3+^ corner in **1** (Fig. 3f), and two Ce^4+^_3_ Ce^3+^ squares in **3** (Fig. 3c) are all bridged by a μ_4_-OH^−^ ion with rare tetragonal pyramidal geometry (the O is 0.7–0.8 Å above the Ce_4_ plane). All μ_4_-OH^−^ ions have similar O BVS values of 0.52–0.71 (Supplementary Tables 3–5), intermediate between those of OH^−^ and H_2_O. In **1** (but not **2** or **3**), the μ_4_-OH^−^ protons (H12 and H14) were observed in difference Fourier maps, confirming them (and by extension those in **2** and **3**) to be OH^−^, not H_2_O. The Ce^4+^··OH^−^and Ce^3+^··OH^−^distances are extremely long (2.7–3.0 Å; Supplementary Table 7) and suggest minimal Ce-O bonding; for comparison, Ce^4+^-μ_3_-O^2−^ = 2.2–2.3 Å, Ce^4+^-μ_4_-O^2−^ = 2.3–2.35 Å, and Ce^4+^-μ_3_-OH^−^ = 2.3–2.45 Å. The very-long Ce··μ_4_-OH^−^ distances suggest an essentially free OH^−^ ion acting as a weakly docked ‘lid’ on the Ce_4_ surface (and thus rationalizing its small BVS). Space-filling representations (Supplementary Fig. 6) show the OH^−^ to be encapsulated by the surrounding carboxylates and cannot move from its μ_4_ central position to become more strongly bound μ_2_ or μ_3_.

**3** also contains planar double-square units (Fig. 3d, e), and these do not contain μ_4_-OH^−^ ions. Instead, those in **3a** (Fig. 3d) have *η* ^2^-carboxylates attached to one Ce that act as lids, tilting inwards so that one O atom approaches the mid-point of each square; the three resulting Ce··O separations (~3.0 Å) indicate extremely weak contacts (Supplementary Table 8). In **3b**, one *η* ^2^-carboxylate of each double-square is replaced by an MeCN, as described above, and this again tilts over the center of the square to act as a lid, giving a very unusual bent binding mode. The three resulting Ce··N separations (>3.0 Å) again indicate only very weak contacts. Interestingly, these planar double squares in **3** are each fused at their Ce^3+^ corners to the μ_4_-OH^−^-bridged Ce^4+^_3_Ce^3+^ squares (Fig. 3c) described above, so that **3** contains two asymmetric L-shaped (86.1°) triple squares with the Ce^3+^ lying at the inner point of the L. For charge balance, **3a** must also contain two additional H^+^. Since the O BVS values indicate they are not on surface μ_3_-O^2−^ ions, we suspected them to be on ligand groups. Indeed, three carboxylate O atoms attached to Ce^3+^, namely O40, O40′ and O83, form a triangle and all show lowered BVS values of 1.78, 1.78 and 1.65, respectively, suggesting an H^+^ is capping each of the two triangles in **3a** by interacting with the O atoms in a trifurcated fashion (Supplementary Table 5). The formulation of **3b** can now be rationalized as resulting from loss of some of the chelating MeCO_2_^−^ groups in **3a**, assisted by protonation to MeCO_2_H by these H^+^, and replacement by MeCN solvent molecules in **3b**.

The two Ce^3+^ each in **1** and **3** are thus all at surface sites, as suggested to also be the case in CNPs^10,38^. The lower Ce^3+^charge favors fewer O^2−^ ligands than Ce^4+^ and thus disfavors body sites. In contrast, **2** contains no Ce^3+^. Interestingly, all Ce^3+^ occur within (100) Ce^4+^_3_Ce^3+^ square facets. In **3** (Fig. 3c), the Ce^3+^··OH^−^ and Ce^4+^··OH^−^ distances are identical (~2.7 Å), again supporting weak contacts by the μ_4_-OH^−^. In **1**, the V-shaped double-square joined at the Ce^3+^corner (Fig. 3f) has a Ce^3+^··OH^−^ distance of ~ 2.7 Å on one side, but this causes a longer Ce^3+^··OH^−^ on the other (~3.0 Å; Supplementary Table 7). It is also extremely interesting that when Ce^3+^ ions are present, the surface H^+^ (i.e., OH^−^ ions, and H^+^ hydrogen bonding to carboxylate groups) are located very close to them. The presence and positions of H^+^ on nanoparticles are extremely challenging to determine^39^, but in nanoclusters **1** and **3** most of them are directly observed and clearly accumulate on O atoms near Ce^3+^ (Fig. 1d, f). The effect is likely synergistic, i.e., the lower Ce^3+^ charge favors accumulation of H^+^ nearby, which in turn mollify the O^2−^ and carboxylate charges and stabilize the lower Ce^3+^ charge. In contrast, with no Ce^3+^ in **2**, the H^+^spread out over the surface (Fig. 1e), although they again favor Ce_4_ squares. H^+^ are expected to be mobile on the nanocluster surfaces, as recent work has concluded from studies of hydrogen mobility (‘hopping’) on surface O atoms of CeO_2_ thin films^40^. Double protonation of an O^2−^ and desorption of surface H_2_O was suggested as the means of forming O vacancies.

## Discussion

We have shown that a bottom-up synthetic approach in solution at ambient temperatures using readily available reagents can be successfully applied to obtain a family of monodisperse metal oxide nanoparticles of ultra-small dimensions. This thus achieves for metal oxides what was previously accomplished for the distinctly different area of metal nanoparticles, particularly of Au. In the present work, monodisperse CeO_2_ nanoclusters with the fluorite structure and monolayer organic ligand shells can be synthesized and structurally characterized to atomic resolution. They exhibit multifaceted structures consisting mainly of (100) and (111) facets, but **3** also has (110) facets giving noticeable surface kinks/edges/trenches. The surface location of any Ce^3+^ ions and the H^+^ positions on μ_3_- and μ_4_-OH^−^ groups, as well as ligand groups, are particularly welcome to know. The μ_4_-OH^−^are weakly attached with long Ce···O distances to the (100) facets, acting as lids on Ce_4_ squares, as also do O (carboxylate) and N (MeCN) lids on other (100) facets. Such surface features are likely of great relevance to CNP reactivity: Under heterogeneous catalysis conditions, or in solution or colloidal suspension, one can envisage the ready loss or ‘opening’ of such weakly interacting lids (e.g., by protonation of OH^−^, detachment of MeCN, or tilting away of the chelating carboxylate, perhaps by becoming monodentate) exposing Ce_4_ square faces for reaction. We thus propose these weakly lidded Ce_4_ sites as resting states of some of the catalytically highly reactive, surface O-vacancy sites in CNPs. In addition, when Ce^3+^ ions are present, their locations in **1** and **3** corner-linking two (100) Ce^3+^Ce^4+^_3_ squares, and the concomitant accumulation nearby of mobile H^+^, on μ_3_-OH^−^, μ_4_-OH^−^ and/or ligand groups, together offer a possible picture for the high catalytic activity of surface Ce^3+^ in CNPs. Similarly, the kinks/edges/trenches associated with the (110) facets in **3** suggest additional sites of increased reactivity, as seen for (110) facets of CNPs, and they have also been identified in CNPs as nucleation sites for heterometals^41,42^, a process we are trying to mimic with **3**. We note that there is a general consensus that the (111) facet of CNPs is the most thermodynamically stable while the (100) facet is highly reactive due its lower stability and is therefore a proposed site for O vacancies and Ce^3+^ ions^17,29,43^, observations that are consistent with the surface features we have identified in **1**–**3**.

Even on the basis of only the three nanoclusters described herein, it is already apparent how CNPs with similar sizes can have very different properties and reactivities. Although **2** and **3** are essentially the same size and metal nuclearity, they differ significantly in their overall shape, the variety of facets they exhibit, the resulting surface morphology, and their Ce^3+^ content. On the other hand, the availability now of samples of identical, monodisperse nanoclusters makes possible the study of activity vs. exact size, surface morphology and Ce^3+^ content. In addition, while dispersions of CNPs in water are often unstable, leading to agglomeration that can affect their transport, distribution and reactivity, particularly for ultra-small CNPs in biomedical studies, **3** is completely water soluble and affords the opportunity to study reactivity in biologically relevant media^27,44^.

Finally, **1**–**3** contain either Ce^4+^_4_-μ_4_-OH^−^ or Ce^3+^Ce^4+^_3_-μ_4_-OH^−^(100) squares, or both, and this variation may also be responsible for the recognized redox-state dependent ROS-scavenging ability and toxicity of CNPs with different amounts of surface Ce^3+^ ^21,22,45,46^. In contrast, recent suggestions that 1.1–3.5 nm CNPs should have a defect-fluorite structure and a large surface Ce^3+^:Ce^4+^ ratio are not supported by **1**–**3** ^47,48^. Ce^3+^ is certainly on the surface, but no correlation between size and the number of Ce^3+^ is seen, with Ce_24_ and Ce_40_ having two each, but Ce_38_ none. In fact, given that Ce^3+^ ions in **1**–**3** always occur at the centre of V-shaped double-square subunits (Fig. 3f and similar), we hypothesize that the higher symmetry, essentially spherical **2** contains no Ce^3+^ because its surface structure contains no such double squares. Furthermore, the conventional wisdom that smaller CNPs have more surface Ce^3+^ may reflect the greater number of such V-shaped units present in smaller nanoparticles of lower symmetry as a result of their increased number of points of high curvature. We are currently seeking to extend the family to larger nanoclusters and higher Ce^3+^:Ce^4+^ ratios, and exploring the reactivity of **1**–**3** with ROS.

## Methods

### Syntheses

[Ce_24_O_28_(OH)_8_(PhCO_2_)_30_(py)_4_] (**1**) was prepared by the reaction of (NH_4_)_2_[Ce(NO_3_)_6_] and PhCO_2_H in a 1:2 molar ratio in pyridine at room temperature. The golden-yellow solution was stirred for 30 mins, diluted with 2 volumes of MeCN, and maintained undisturbed for 1 week. The resulting yellow square plates of **1**∙9py were collected by filtration, washed with MeCN, and dried in vacuum. The yield was 14% based on Ce. Anal. Calcd (Found) for dried **1**∙2py (C_240_H_188_Ce_24_N_6_O_96_): C, 35.79 (35.63); H, 2.35 (2.00); N, 1.04 (0.98).

[Ce_38_O_54_(OH)_8_(EtCO_2_)_36_(py)_8_] (**2**) and[Ce_40_O_56_(OH)_2_(MeCO_2_)_44_(MeCO_2_H)_2_(py)_4_]/ [Ce_40_O_56_(OH)_2_(MeCO_2_)_44_(MeCN)_2_(py)_4_](**3**) were prepared by the reactionof (NH_4_)_2_[Ce(NO_3_)_6_], the corresponding RCO_2_H, and NH_4_I in a 1:4:1 molar ratio in pyridine/H_2_O (10:1 v/v) at room temperature. The golden-yellow solutions were stirred for 30 min, diluted with two volumes of MeCN, and maintained undisturbed for 4 weeks. The resulting yellow square plates (**2**∙16MeCN) or rods (**3**∙48MeCN) were collected by filtration, washed with MeCN, and dried in vacuum. The yields were 49% and 35% for **2** and **3**, respectively. Anal. Calcd (Found) for **2**∙7H_2_O (C_148_H_242_Ce_38_N_8_O_141_): C, 18.30 (17.90); H, 2.51 (2.36); N, 1.15 (1.05). Anal. Calcd (Found) for **3∙**8H_2_O (C_112_H_178_Ce_40_N_5_O_156_): C, 13.88(13.79); H, 1.85 (1.78); N, 0.72 (0.76). The indicated atomic composition of **3** is calculated using the average of the **3a** and **3b** formulas.

Fuller details of the three syntheses and infra-red spectral data for **1**–**3** are available in Supplementary Methods.

### X-ray crystallography

Single-crystal X-ray diffraction studies at −173 °C were performed on a Bruker DUO diffractometer using MoK_α_ (*λ* = 0.71073 Å) or CuK_α_ (*λ* = 1.54178 Å) radiation (from an ImuS power source), and an APEXII CCD area detector (Supplementary Methods and Supplementary Table 1). The metric parameters of the refined structures were used to determine the Ce oxidation states and the O protonation levels by bond valence sum (BVS) calculations (Supplementary Tables 2–5).

### Data availability

The crystallographic information files (CIFs) for [Ce_24_O_28_(OH)_8_(PhCO_2_)_30_(py)_4_]∙9py (**1**∙9py), [Ce_38_O_54_(OH)_8_(EtCO_2_)_36_(py)_8_]∙16MeCN (**2**∙16MeCN), and [Ce_40_O_56_(OH)_2_(MeCO_2_)_44_(MeCO_2_H)_2/0_(MeCN)_0/2_(py)_4_]∙48MeCN (**3**∙48MeCN) have been deposited at the Cambridge Crystallographic Data Centre with deposition codes CCDC 1529955-1529957 for **1**–**3**, respectively.

## Electronic supplementary material

Supplementary Information
