# Supplementary material for: Atomically-precise colloidal nanoparticles of cerium dioxide
Source: Nat Commun. 2017 Nov 13;8:1445. doi: 10.1038/s41467-017-01672-4 (PMC5682284; doi:10.1038/s41467-017-01672-4)
Supplement: Supplementary file 1 — Supplementary Information [file 41467_2017_1672_MOESM1_ESM.pdf]

Supplementary Methods

All the syntheses below have been reproduced many times, giving yields in a range within a few percent of the quoted yield. The NH_4I was added to provide a weak reducing agent to readily allow formation of Ce^{3+}, should the reaction product so desire. The yields are not optimized: at this stage, we have targeted well-formed crystalline solids from slow crystallizations to allow definitive characterization by single-crystal X-ray crystallography, rather than faster precipitation of products as powders.

$[Ce_{24}O_{28}(OH)_8(PhCO_2)_{30}(py)_4]$ (**1**). To a stirred solution of pyridine (10 mL) was added $(NH_4)_2[Ce(NO_3)_6]$ (0.55 g, 1.0 mmol) and $PhCO_2H$ (0.25 g, 2.0 mmol). The golden yellow solution was stirred for 30 minutes, followed by the addition of 20 mL of MeCN. The solution was then left to sit for one week, during which time X-ray quality yellow square plates of **1**·9py formed. They were collected by filtration, washed with MeCN, and dried in vacuum. The yield was 14% based on Ce. Anal. Calcd (Found) for dried **1**·2py ($C_{240}H_{188}Ce_{24}N_6O_{96}$): C, 35.79 (35.63); H, 2.35 (2.00); N, 1.04 (0.98). Selected IR data (cm^{-1}): 3435 (b), 3059 (w), 1594 (m), 1534 (s), 1492 (w), 1447 (w), 1402 (s), 1307 (w), 1178 (m), 1069 (m), 1025 (m), 849 (w), 717 (s), 688 (w), 672 (w), 565 (m), 506 (m), 431 (s).

$[Ce_{38}O_{54}(OH)_8(EtCO_2)_{36}(py)_8]$ (**2**). To a stirred solution of aqueous pyridine (11 mL, 10:1 v/v) was added $(NH_4)_2[Ce(NO_3)_6]$ (0.55 g, 1.0 mmol) and propionic acid (0.30 mL, 4.0 mmol) followed by NH_4I (0.15 g, 1.0 mmol). The golden yellow solution was stirred for 30 minutes, followed by the addition of 20 mL of MeCN. The solution was then left to sit for four weeks, during which time X-ray quality yellow square plates of **2**·16MeCN formed. They were collected by filtration, washed with MeCN, and dried in vacuum. The yield was 49% based on Ce. Anal. Calcd (Found) for **2**·7H_2O ($C_{148}H_{242}Ce_{38}N_8O_{141}$): C, 18.30 (17.90); H, 2.51 (2.36); N, 1.15 (1.05). Selected IR data (cm^{-1}): 3570 (w), 3432 (b), 3175 (b), 2975 (m), 2940 (m), 2879 (w), 1575 (w), 1537 (s), 1465 (m), 1415 (s), 1371 (m), 1295 (m), 1277 (m), 1078 (m), 1056 (w), 1005 (w), 891 (m), 814 (m), 698 (w), 567 (w), 492 (s). Although **2** contains no Ce^{3+}, the NH_4I was essential to yield pure product.

$[Ce_{40}O_{56}(OH)_2(MeCO_2)_{44}(MeCO_2H)_{2/0}(MeCN)_{0/2}(py)_4]$ (**3**). To a stirred solution of aqueous pyridine (11 mL, 10:1 v/v) was added $(NH_4)_2[Ce(NO_3)_6]$ (0.55 g, 1.0 mmol) and acetic acid (0.23 mL, 4.0 mmol) followed by NH_4I (0.15 g, 1.0 mmol). The golden yellow solution was stirred for 30 minutes, followed by the addition of 20 mL of MeCN. The solution was then left to sit for four weeks, during which time X-ray quality yellow square rods of **3**·48MeCN formed. They were collected by filtration, washed with MeCN, and dried in vacuum. The yield was 35% based on Ce. Anal. Calcd (Found) for **3**·8H_2O ($C_{112}H_{177}Ce_{40}N_5O_{156}$): C, 13.88 (13.79); H, 1.84 (1.78); N, 0.72 (0.76). The indicated atomic composition of **3** is calculated using the average of the **3a** and **3b** formulas. Selected IR data (cm^{-1}): 3408 (b), 3171 (b), 1540 (s), 1400 (s), 1385 (s), 1335 (m), 1050 (w), 1019 (w), 940 (w), 671 (w), 613 (w), 513 (s), 434 (m).

Single-Crystal X-ray Crystallography

Crystallographic information files have been deposited at the Cambridge Crystallographic Data Centre with deposition codes **CCDC 1529955-1529957** for **1-3**, respectively.

Crystal structure data for **1**·9py: X-Ray Intensity data for **1** were collected on a Bruker DUO diffractometer using CuKα radiation (λ = 1.54178 Å), from an ImuS power source, and an APEXII CCD area detector. The asymmetric unit consists of a half Ce_{24} cluster and four and a half pyridine solvent molecules. Two of the pyridines are hydrogen-bonded to the cluster (one of them is refined with 50% occupancy) but the other three were significantly disordered and could not be modeled properly. Thus program SQUEEZE[1] a part of the PLATON[2] package of crystallographic software, was used to calculate the solvent disorder area and remove its contribution to the overall intensity data. Four of the O atoms (of the asymmetric half cluster) were protonated and their H atoms were located from a difference Fourier map. Two of them, H12 and H15 were refined riding on their parent atoms while the other two, H14 and H16, were refined freely. There are six disordered phenyl rings and each one was refined in two parts and constrained to maintain an ideal hexagonal geometry. Carbon atoms on one disordered phenyl ring, on C51', had their displacement parameters kept equivalent using command EADP in the SHELX refinement. Similarly, the disordered pyridine solvent was refined in two positions with 60/40% occupancy and idealized geometry as well as constrained displacement parameters using the EADP command.

Crystal structure data for **2**·16MeCN: X-Ray Intensity data for **2** were collected on a Bruker DUO diffractometer using MoKα radiation (λ = 0.71073 Å) and an APEXII CCD area detector. The asymmetric unit consists of two ¼ Ce_{38} clusters and eight MeCN solvent molecules. The solvent molecules were disordered and could not be modeled properly, thus program SQUEEZE,[1] a part of the PLATON[2] package of crystallographic software, was used to calculate the solvent disorder area and remove its contribution to the overall intensity data. Each of the clusters is located on independent 2/m symmetry sites. At the intersection of each cluster, and specifically at the side with four Ce centers forming a square, there are four propionate ligands disordered over eight positions. Similar disordered regions are found in regions 90° from the previous disorders. Application of similar distances, SADI, was implemented in three ligands and EADP to two sets of bonded atoms.

Crystal structure data for **3**·48MeCN: X-Ray Intensity data for **3** were collected on a Bruker DUO diffractometer using MoKα radiation (λ = 0.71073 Å) and an APEXII CCD area detector. The asymmetric unit consists of two ¼ Ce_{40} clusters and twelve acetonitrile solvent molecules. The solvent molecules were disordered and could not be modeled properly, thus program SQUEEZE,[1] a part of the PLATON[2] package of crystallographic software, was used to calculate the solvent disorder area and remove its contribution to the overall intensity data. Each of the clusters is located on independent 2/m symmetry sites. At the intersection of each cluster, and

specifically at the side with four Ce centers forming a square, there are four acetate ligands disordered over eight positions. Similar disordered regions are found in regions 90° from the previous disorders. A major difference between the two independent clusters is that one has two bound MeCN ligands replacing two acetic acid ligands. Application of similar distances, SADI, was implemented in five ligands.

Supplementary Note 1

When studying/analyzing large clusters containing heavy atoms such as cerium, the intensity of reflections is usually dominated by the heavy metal atoms. Thus information about light atoms is usually of lower quality than those of the core of the metal cluster. A higher effect is also observed in the positions of disordered light atom solvents. In all of these structures, larger-than-usual observed residual positive electron density is always observed at the end of the full refinement. Data truncation is also a major contributor to observing larger residual positive electron density.

Supplementary Table 1. Crystal Data and Structure Refinement Parameters for **1**, **2**, and **3**.

	**1**·9py	**2**·16MeCN	**3**·48MeCN
formula[a]	$C_{230}H_{178}Ce_{24}N_4O_{96}$	$C_{148}H_{228}Ce_{38}N_8O_{134}$	$C_{112}H_{161}Ce_{40}N_5O_{148}$[b]
Fw, g mol^{-1}	7896.77	9587.92	9550.20
space group	*P2₁/n*	*C2/m*	*P2/m*
a, Å	21.2942(6)	29.205(2)	17.4795(7)
b, Å	25.0588(7)	28.175(2)	21.0019(9)
c, Å	25.4417(7)	29.809(2)	38.6269(16)
α, deg	90	90	90
β, deg	91.9276(14)	92.2675(15)	98.254(1)
γ, deg	90	90	90
V, Å^3	13568.2(7)	24509(3)	14033.2(10)
Z	2	4	1
T, K	100(2)	108(2)	100(2)
λ, Å[c]	1.54178 Å	0.71073	0.71073
ρ_{calc}, g cm^{-3}	2.030	2.596	2.492
μ, mm^{-1}	31.111	6.988	6.433
$R1$[d, e]	0.0554	0.0768	0.0626
$wR2$[f]	0.1580	0.1956	0.1884

[a] Excluding solvent molecules of crystallization. [b] Average of the formulas of **3a** and **3b**. [c] Graphite monochromator. [d] $I > 2\sigma(I)$. [e] $R1 = 100\Sigma(||F_o| - |F_c||)/\Sigma|F_o|$. [f] $wR2 = 100[\Sigma [w(F_o^2 - F_c^2)^2]/ \Sigma[w(F_o^2)^2]]^{1/2}$, $w = 1/[\Sigma^2(F_o^2) + [(ap)^2 + bp]$, where $p = [\max (F_o^2, 0) + 2F_c^2]/3$.

Supplementary Table 2. Bond Valence Sums and Coordination Numbers (CN) for Ce Atoms in **1**, **2**, and **3**.[a, b]

**1**			
Atom	CN	Ce^{III}	Ce^{IV}
Ce1	9	4.33	**3.81**
Ce2	9	4.28	**3.76**
Ce3	9	4.46	**3.92**
Ce4	10	**3.01**	2.65
Ce5	8	4.28	**3.76**
Ce6	8	4.54	**3.99**
Ce7	9	4.29	**3.77**
Ce8	8	4.46	**3.91**
Ce9	9	4.30	**3.77**
Ce10	8	4.37	**3.84**
Ce11	8	4.24	**3.72**

**2a**[c]				**3a**			
Atom	CN	Ce^{III}	Ce^{IV}	Atom	CN	Ce^{III}	Ce^{IV}
Ce1	9	4.29	**3.76**	Ce1	9	4.39	**3.86**
Ce2	8	4.45	**3.91**	Ce2	8	4.37	**3.84**
Ce3	9	4.39	**3.86**	Ce3	9	4.39	**3.85**
Ce4	8	4.61	**4.06**	Ce4	8	4.46	**3.92**
Ce5	8	4.62	**4.06**	Ce5	8	4.63	**4.07**
Ce6	9	4.36	**3.83**	Ce6	8	4.43	**3.89**
Ce7	8	4.39	**3.85**	Ce7	8	4.31	**3.79**
Ce8	9	4.27	**3.75**	Ce8	9	4.33	**3.81**
Ce9	9	4.33	**3.80**	Ce9	9	4.58	**4.02**
Ce10	8	4.43	**3.89**	Ce10	8	4.25	**3.74**
Ce11	9	4.71	**4.13**	Ce11	8	4.21	**3.70**
				Ce12	7	4.36	**3.83**
				Ce13	10	**2.81**	2.47

**2b**[c]				**3b**			
Atom	CN	Ce^{III}	Ce^{IV}	Atom	CN	Ce^{III}	Ce^{IV}
Ce21	9	4.19	**3.68**	Ce21	8	4.41	**3.87**
Ce22	8	4.45	**3.91**	Ce22	7	4.24	**3.73**
Ce23	9	4.35	**3.82**	Ce23	9	4.34	**3.81**
Ce24	9	4.18	**3.67**	Ce24	8	4.06	**3.56**
Ce25	8	4.50	**3.95**	Ce25	8	4.66	**4.09**
Ce26	8	4.64	**4.07**	Ce26	8	4.31	**3.78**
Ce27	9	4.51	**3.96**	Ce27	8	4.14	**3.64**
Ce28	8	4.50	**3.95**	Ce28	8	4.23	**3.71**
Ce29	8	4.55	**4.00**	Ce29	8	4.47	**3.93**
Ce30	9	4.52	**3.97**	Ce30	9	4.39	**3.86**
Ce31	9	4.54	**3.98**	Ce31	10	**3.20**	2.81
Ce32	9	4.84	**4.25**	Ce32	8	4.46	**3.91**
Ce33	9	4.87	**4.27**	Ce33	9	4.49	**3.95**

[a] The bold values are the ones closest to the charge for which they were calculated; the oxidation state is thus the nearest integer to the bold value. [b] CN = coordination number. [c] The formulas of **2a** and **2b** are identical.

Supplementary Table 3. Bond Valence Sums and Assignments for the O Atoms[a] in **1**.

**1**	Atom	BVS	Assignment
	O1	1.89	O^{2-}
	O2	1.94	O^{2-}
	O3	1.88	O^{2-}
	O4	2.09	O^{2-}
	O5	2.15	O^{2-}
	O6	1.94	O^{2-}
	O7	2.17	O^{2-}
	O8	1.81	O^{2-}
	O9	2.08	O^{2-}
	O10	2.09	O^{2-}
	O11	2.11	O^{2-}
	O12	0.71	μ_4-OH^- [b, c]
	O13	1.94	O^{2-}
	O14	0.69	μ_4-OH^- [b, c]
	O15	1.21	μ_3-OH^- [b]
	O16	1.21	μ_3-OH^- [b]
	O17	1.94	O^{2-}
	O19	1.71	O^{2-}

[a] An oxygen BVS in the ~1.8-2.0, ~0.9-1.2 and ~0.2-0.4 ranges is indicative of non-, single- and double- protonation, respectively. [b] Two by symmetry. [c] Square pyramidal O geometry.

Supplementary Table 4. Bond Valence Sums and Assignments for the non-Carboxylate O Atoms[a] in **2**.

**2a**	Atom	BVS	Assignment	**2b**	Atom	BVS	Assignment
	O1	1.93	O^{2-}		O42	0.52	μ_4-OH^- [b,d]
	O3	1.86	O^{2-}		O45	2.12	O^{2-}
	O4	2.14	O^{2-}		O49	1.65	O^{2-} [c,e]
	O7	0.57	μ_4-OH^- [b,d]		O50	2.12	O^{2-}
	O9	2.13	O^{2-}		O54	2.11	O^{2-}
	O10	2.11	O^{2-}		O59	1.86	O^{2-}
	O11	2.00	O^{2-}		O60	1.72	O^{2-} [c,e]
	O12	2.04	O^{2-}		O63	2.03	O^{2-}
	O13	2.11	O^{2-}		O64	0.57	μ_4-OH^- [b,d]
	O14	1.96	O^{2-}		O69	1.91	O^{2-}
	O15	2.11	O^{2-}		O70	2.15	O^{2-}
	O16	1.90	O^{2-}		O71	2.10	O^{2-}
	O17	2.12	O^{2-}		O72	1.99	O^{2-}
	O18	1.71	O^{2-} [b,e]		O73	2.12	O^{2-}
	O27	0.57	μ_4-OH^- [c,d]		O74	1.96	O^{2-}
	O28	2.10	O^{2-}		O75	0.58	μ_4-OH^- [b,d]
	O29	1.86	O^{2-}		O79	1.89	O^{2-}
	O30	1.91	O^{2-}				
	O39	1.52	O^{2-} [b,e]				

[a] An oxygen BVS in the ~1.8-2.0, ~0.9-1.2 and ~0.2-0.4 ranges is indicative of non-, single- and double- protonation, respectively. [b] Two by symmetry. [c] Four by symmetry. [d] Square pyramidal O geometry. [e] Possible partial occupancy by OH^-.

Supplementary Table 5. Bond Valence Sums and Assignments for the O Atoms[a] in **3**.

**3a**	Atom	BVS	Assignment	**3b**	Atom	BVS	Assignment
	O1	2.14	O^{2-}		O41	1.95	O^{2-}
	O2	2.09	O^{2-}		O42	2.09	O^{2-}
	O4	0.64	μ_4-OH^- [b]		O43	2.07	O^{2-}
	O5	2.00	O^{2-}		O44	2.00	O^{2-}
	O6	1.98	O^{2-}		O45	2.00	O^{2-}
	O7	1.88	O^{2-}		O46	2.00	O^{2-}
	O8	2.06	O^{2-}		O47	2.07	O^{2-}
	O9	2.10	O^{2-}		O48	2.18	O^{2-}
	O10	2.06	O^{2-}		O49	1.90	O^{2-}
	O11	2.01	O^{2-}		O50	1.97	O^{2-}
	O12	1.88	O^{2-}		O51	1.85	O^{2-}
	O13	1.99	O^{2-}		O52	1.86	O^{2-}
	O14	1.94	O^{2-}		O53	1.95	O^{2-}
	O15	1.94	O^{2-}		O54	2.14	O^{2-}
	O16	1.99	O^{2-}		O55	1.98	O^{2-}
	O17	2.06	O^{2-}		O57	0.64	μ_4-OH^- [b]
	O40	1.78	RO^- [c]				
	O40′	1.78	RO^- [c]				
	O83	1.65	RO^- [c]				

[a] An oxygen BVS in the ~1.8-2.0, ~0.9-1.2 and ~0.2-0.4 ranges is indicative of non-, single- and double- protonation, respectively. [b] Square pyramidal O geometry. [c] Carboxylate O atoms forming a triangle that is proposed to be capped by a H^+ giving lowered BVS values for the O atoms akin to partial occupancy by ROH (i.e., $MeCO_2H$). A prime indicates the symmetry-related atom.

Supplementary Table 6. Bond distances for μ_3-OH^- in a triangle of Ce^{4+} ions.

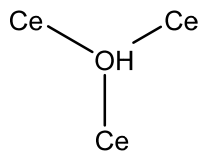

	Parameter	Distance (Å)
**1**	Ce2-O15	2.364(5)
	Ce7-O15	2.411(5)
	Ce10-O15	2.450(5)
	Ce3-O16	2.367(5)
	Ce8-O16	2.429(5)
	Ce9-O16	2.420(5)
**2a**	Ce7-O39	2.295(15)
	Ce11-O39	2.34(7)
	Ce11′-O39	2.34(7)

Supplementary Table 7. Ce-O separations in μ_4-OH^- -bridged Ce^{3+}/$3Ce^{4+}$ and $4Ce^{4+}$ squares

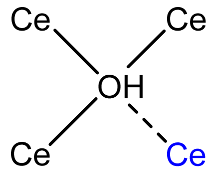

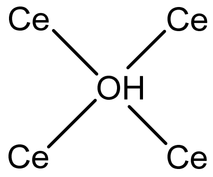

	Parameter	Distance (Å)
**1**	Ce1-O12	2.843(6)
	Ce2-O12	2.674(5)
	Ce3-O12	2.669(5)
	Ce4-O12	2.724(6)
	Ce4-O14	2.990(6)
	Ce7-O14	2.600(5)
	Ce9-O14	2.681(5)
	Ce12-O14	2.721(6)
**3a**	Ce1-O4	2.739(13)
	Ce8-O4	2.766(4)
	Ce8′-O4	2.766(4)
	Ce13-O4	2.766(13)
**3b**	Ce30-O57	2.755(3)
	Ce30′-O57	2.755(3)
	Ce31-O57	2.751(14)
	Ce33-O57	2.736(14)

	Parameter	Distance (Å)
**2a**	Ce1-O7	2.808(13)
	Ce1′-O7	2.808(13)
	Ce8-O7	2.772(13)
	Ce8′-O7	2.772(13)
	Ce3-O27	2.816(13)
	Ce6-O27	2.743(12)
	Ce9-O27	2.861(12)
	Ce11-O27	2.750(12)
**2b**	Ce23-O64	2.790(17)
	Ce27-O64	2.790(5)
	Ce32-O64	2.795(17)
	Ce27-O64	2.790(5)
	Ce30-O75	2.796(5)
	Ce31-O75	2.711(16)
	Ce33-O75	2.846(16)
	Ce30-O75	2.796(5)
	Ce21-O42	2.815(5)
	Ce24-O42	2.832(5)
	Ce21-O42	2.815(5)
	Ce24-O42	2.832(5)

Supplementary Table 8. Ce-O/N separations in RCO_2^-- and MeCN-bridged Ce^{3+}/3Ce^{4+} and 4Ce^{4+} squares

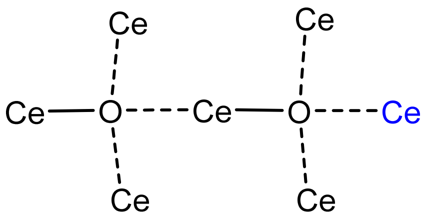

	Parameter	Distance (Å)
**3a**	Ce9-O32	2.715(13)
	Ce10-O32	2.996(41)
	Ce10-O32	2.996(41)
	Ce11-O32	3.054(11)
	Ce11-O38	2.628(13)
	Ce12-O38	3.004(43)
	Ce12-O38	3.004(43)
	Ce13-O38	3.388(12)

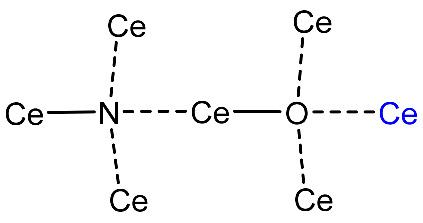

	Parameter	Distance (Å)
**3b**	Ce32-N3	2.69(3)
	Ce27-N3	3.098(13)
	Ce27-N3	3.098(13)
	Ce24-N3	3.269(28)
	Ce24-O74	2.535(16)
	Ce22-O74	3.045(72)
	Ce22-O74	3.045(72)
	Ce31-O74	3.483(15)

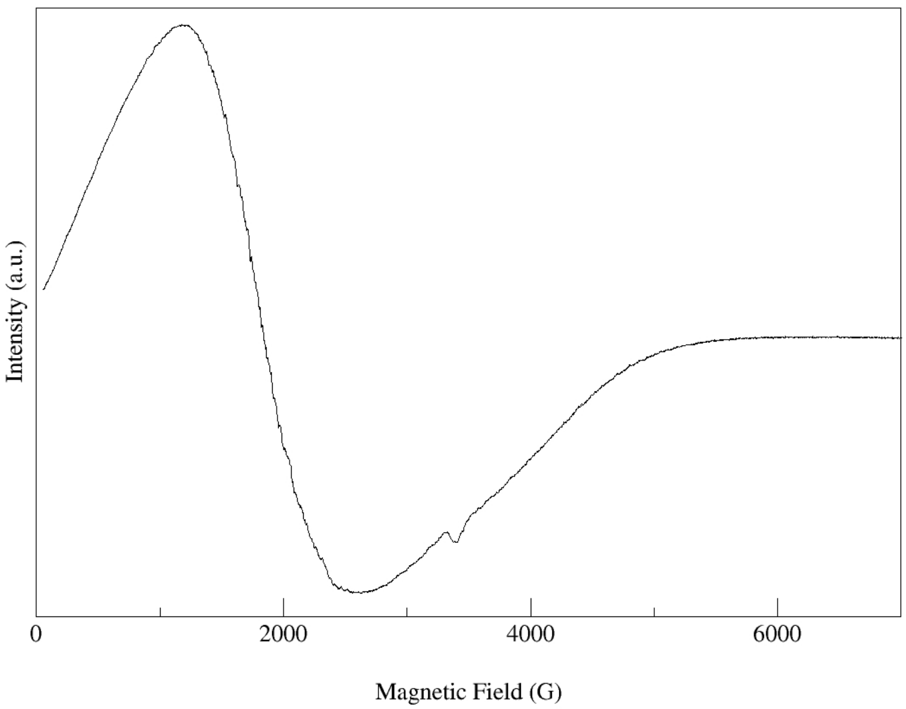

Supplementary Figure 1. EPR spectrum of Ce_{24} nanocluster **1** as a microcrystalline powder at 5.0 K in the 0 to 7000 G field range. The signal around 3500 G is from a Cu impurity present in the resonator.

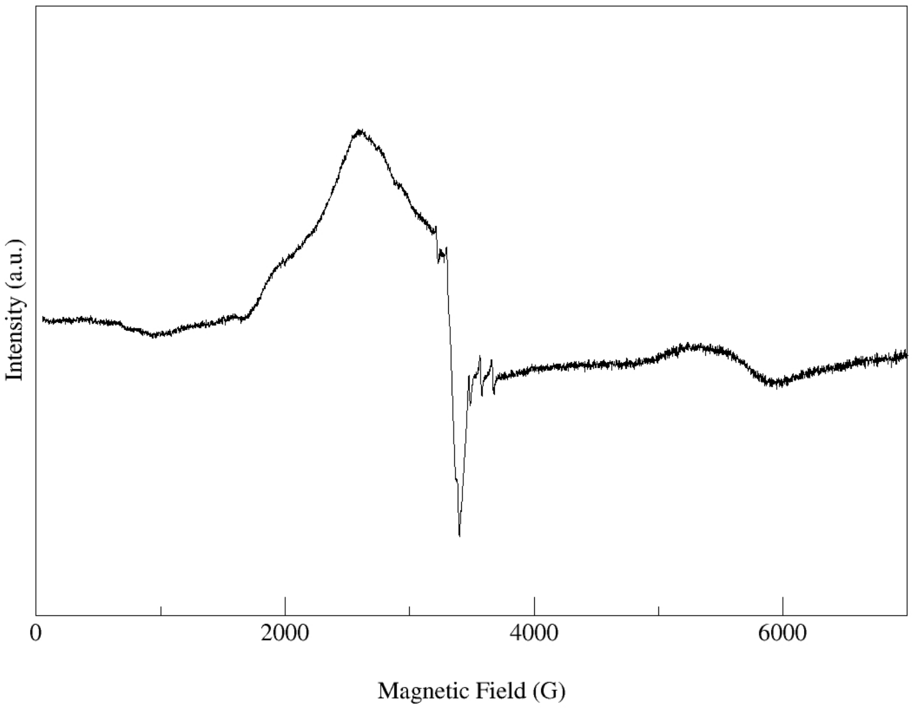

Supplementary Figure 2. EPR spectrum of Ce_{40} nanocluster **3** as a microcrystalline powder at 5.0 K in the 0 to 7000 G field range. The large signal around 3500 G is from a Cu impurity present in the resonator.

	(100)	(110)	(111)
**bulk CeO_2**	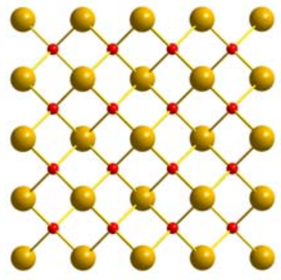	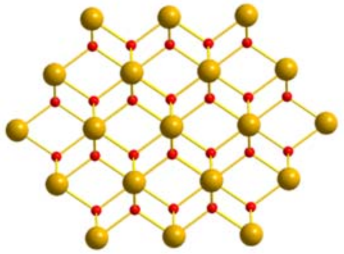	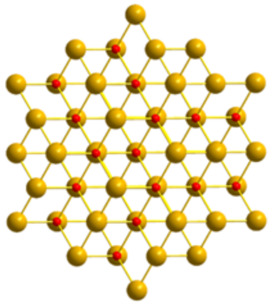
**3a**	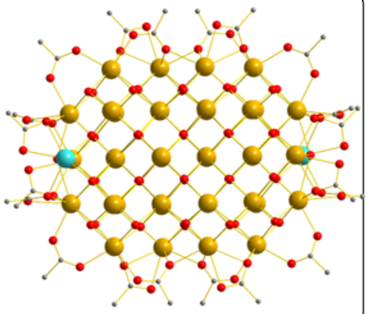	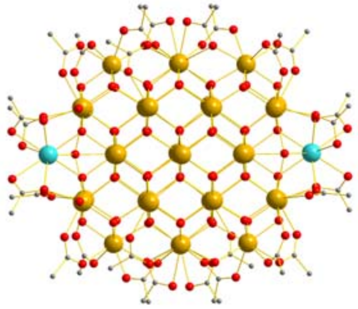	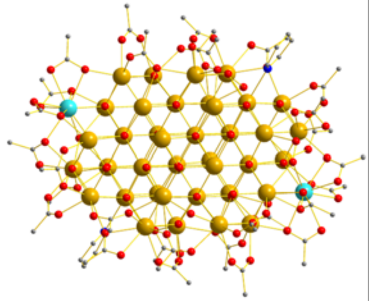

Supplementary Figure 3. Comparison of the core faces of Ce_{40} nanocluster **3a** with those of bulk CeO_2. Three views of **3a** showing the correspondence with the (100), (110), and (111) faces of CeO_2. Colour code: Ce^{4+} gold, Ce^{3+} sky-blue, O red, C grey.

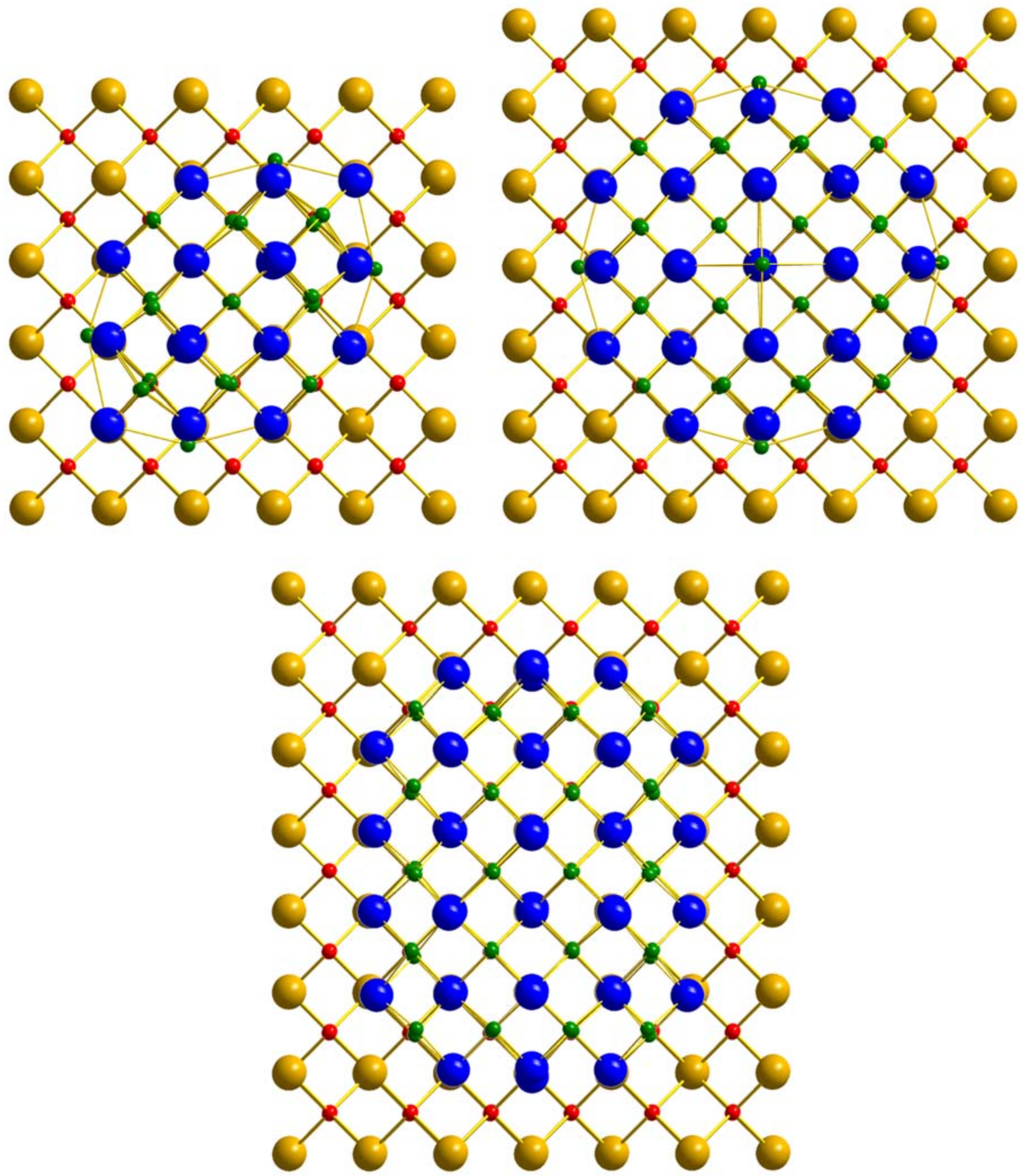

Supplementary Figure 4. Overlays of the Ce/O cores of **1-3** (Ce blue, O green) on the CeO_2 structure (Ce gold, O red) showing only a small deviation of the nanocluster core atoms from their positions in bulk CeO_2.

a)

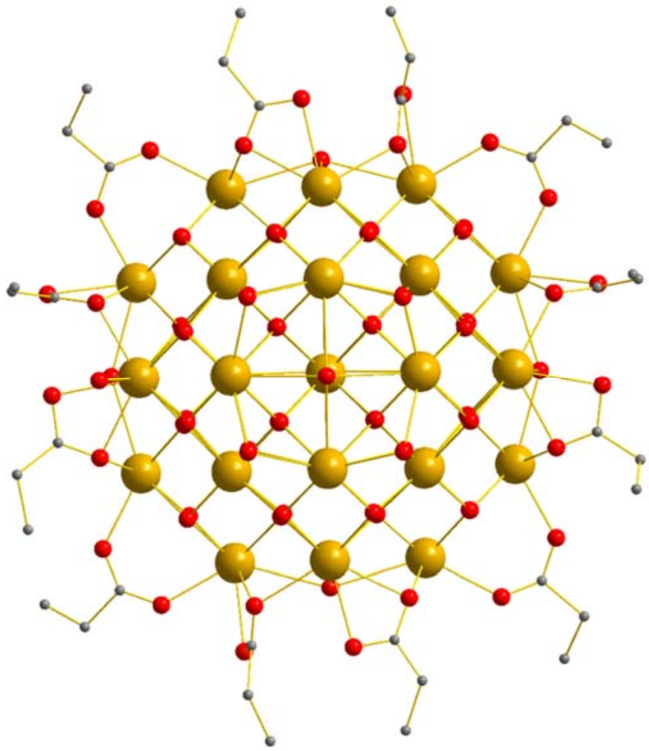

b)

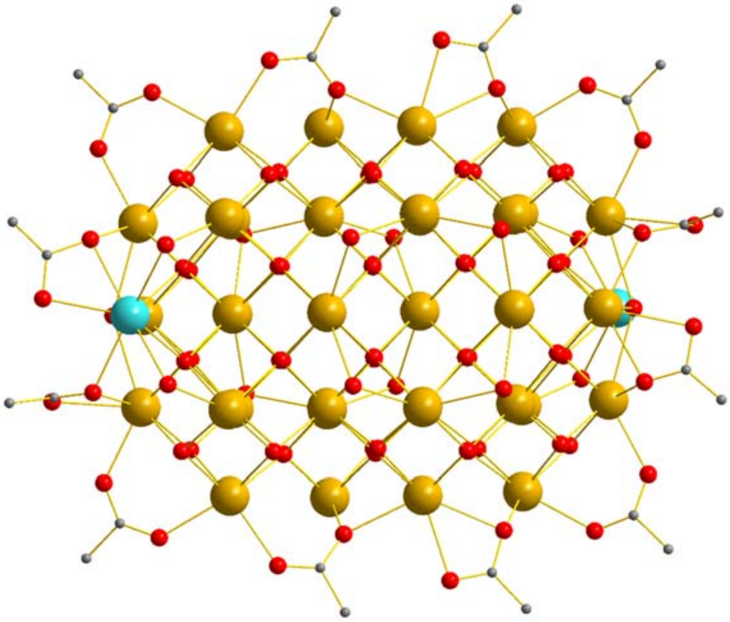

Supplementary Figure 5. Slices from the structures of a) Ce_{38} **2** and b) Ce_{40} **3a**, chosen to emphasize how the versatile and flexible binding modes of carboxylates allow them to accommodate points of high surface curvature, as well as binding to either Ce2 or Ce3 units with differing metal separations.

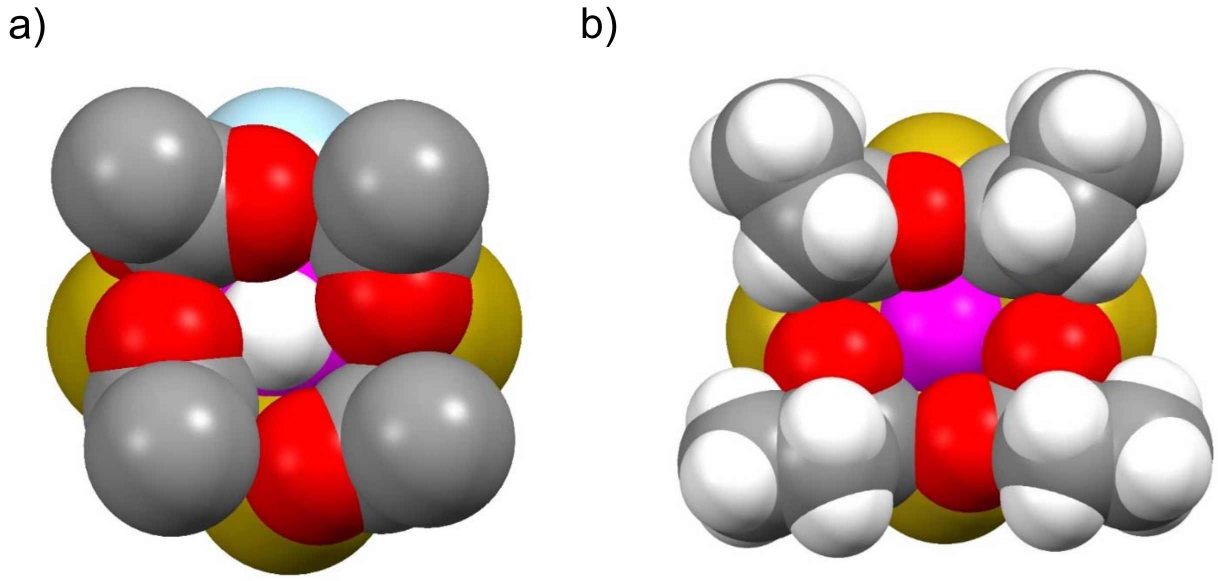

Supplementary Figure 6. Space-filling representations of **(a)** the {$Ce^{3+}Ce^{4+}{}_3(\mu_4\text{-OH})$} unit of Ce_{24} nanocluster **1**, and **(b)** one {$Ce^{4+}{}_4(\mu_4\text{-OH})$} unit of Ce_{38} nanocluster **2**. Both are viewed approximately perpendicular to the Ce_4 planes, emphasizing the μ_4-OH^- at the centre and the surrounding carboxylate ligands; for clarity in **1**, only the carboxylate and ipso C atoms of the benzoate phenyl rings are shown. Colour code: Ce^{4+} gold, Ce^{3+} sky-blue, μ_4-O violet, other O red, C grey, H white.

Supplementary References

1. van der Sluis, P. & Spek, A. L. SQUEEZE, *Acta Cryst*. **A46**, 194-201(1990).
2. Spek, A.L. PLATON, *Acta Cryst*. **D65**, 148-155 (2009).
